# The Preparation and Characterization of MnFe_2_O_4_-Decorated Expanded Graphite for Removal of Heavy Oils from Water

**DOI:** 10.3390/ma12121913

**Published:** 2019-06-13

**Authors:** Hoang Doan Tuan Nguyen, Hoang Tung Nguyen, Thuong Thi Nguyen, Ai Kha Le Thi, Thanh Duy Nguyen, Quynh Thi Phuong Bui, Long Giang Bach

**Affiliations:** 1 NTT Hi-Tech Institute, Nguyen Tat Thanh University, 755000 Ho Chi Minh City, Vietnam; ndth1997@gmail.com; 2 Faculty of Chemical Engineering, Ho Chi Minh City University of Food Industry, 705800 Ho Chi Minh City, Vietnam; aikhale25@gmail.com (A.K.L.T.); thanhduy0525@gmail.com (T.D.N.); phuongquynh102008@gmail.com (Q.T.P.B.); 3 Institute of Fundamental and Applied Sciences, Duy Tan University, 700000 Ho Chi Minh City, Vietnam; nguyen.hoang.tung.13122@gmail.com; 4 Center of Excellence for Green Energy and Environmental Nanomaterials, Nguyen Tat Thanh University, 755000 Ho Chi Minh City, Vietnam

**Keywords:** expanded graphite, heavy oils, magnetic adsorbent, manganese ferrite, wastewater treatment

## Abstract

Recently, many methods have been developed to efficiently eliminate oil spills due to its long-term harmful effects on marine life and human health. Expanded graphite (EG) has been considered as an excellent platform to remove contaminated oil from aqueous solution through a facile adsorption route. As an innovative approach, the decoration of magnetic components, namely, MnFe_2_O_4_, into graphite layers was taken into account for facilitating phase separation under magnetic field which resulted into an easy collection of the used adsorbents in a large scale. The expanded graphite/manganese ferrite composites were prepared from Vietnamese graphite flakes via a two-stage process. Characterization was performed using Scanning Electron Microscope (SEM), Fourier-Transform Infrared Spectroscopy (FTIR), X-Ray Powder Diffraction (XRD), Vibrating Sample Magnetometer (VSM), Energy-Dispersive X-ray (EDS), and nitrogen adsorption/desorption analysis. The adsorption behavior of EG-MnFe_2_O_4_ for widespread used heavy oils, including diesel oil and crude oil, was investigated under the effects of adsorption conditions, i.e., contact time, loaded oil dosage, and salinity of mixing oil and water. The obtained results showed successful incorporation of MnFe_2_O_4_ into graphite sheets and no considerable change on the worm-like structure of EG. The results also showed that incorporated manganese ferrites enhanced the magnetism EG up to 16 emu/g, which made the recovery of used adsorbent conveniently. The EG-MnFe_2_O_4_ adsorbents exhibited the strong adsorption ability toward diesel oil (32.20 ± 0.46 g DO/g EG) and crude oil (33.07 ± 0.33 g CO/g EG). In brief, EG-MnFe_2_O_4_ material provides a potential and promising platform with high performance for oil spill removal.

## 1. Introduction

In the past decades, the number of oil spill accidents that occurred during production, transportation, and consumption process has increasingly grown because of the increasing demand for petroleum-based products. The water pollution from oil spill accidents has adversely affected the marine environment and living organism due to hydrocarbon and trace metals (i.e., lead, cadmium, chrome, copper, etc.) contained in oil component [1]. Furthermore, the oil-contaminated water has caused a detrimental impact on human health; for instance, some symptoms often occur when exposed, such as malaise, headache, nausea, sore eyes, diarrhea, itchy skin, etc. [2,3]. Rather use, numerous studies have been conducted to search for an efficient solution for removing heavy oil. There are several methods to address oil pollution, namely biological, chemical, and physical treatment. Among such methods, the adsorption is most favored on the ground of cost-efficiency, facile operation, efficiency, and feasibility for practical application.

There are a variety of adsorbents developed from various kinds of agricultural by-products, e.g., fruit peels, sugar cane bagasse, and rice husk for oil removal [4,5,6]. However, their uses are quite limited due to low adsorption capacity for heavy oils. Therefore, the development of efficient and selective adsorbent to purify the oil-contaminated water is an instrumental task. Expanded graphite (EG) owing to mesoporous structure, large surface area, and float on the water surface has been considered as a promising adsorbent for removing petroleum-based products from aqueous solution [7,8]. Among proposed techniques to exfoliate graphite layers, preparation of EG by using H_2_O_2_ as an oxidant agent has been recognized as a friendly and safe method for the environment, and it does not utilize oxidizing agents, which may lead to contamination, namely nitric acid and substances containing dichromate [9]. Although many advantages of EG have been found in oil removal, EG’s small particle size and low-density cause difficulties in the collection and regeneration of adsorbents after use from aqueous solution in the real marine environment. Thus, it is necessary to introduce magnetism on EG to easily collect and control oil-absorbing material under the effect of the weather condition.

The combination of EG with magnetic particles has recently been considered as an innovative approach for purifying oil-contaminated water. Many researchers have reported the exfoliated graphite decorated by magnetic nanoparticles, providing the sufficient and desired properties of the magnetic adsorbents [10,11,12,13]. For instance, the addition of α-Fe_2_O_3_ nanoparticles into EG via the sol-gel method and subsequently self-combustion technique showed the magnetism value of 13 emu/g despite the uneven distribution of such pentahedron nanoparticles on the surface of EG [14]. For further improvability, Lutfullin et al. reported the effect of modification of EG with particles containing iron phases at high temperature in air and argon environment [10]. The results showed that the incorporation of such particles caused the reduction of adsorption capacity toward liquid hydrocarbons of the as-prepared composites (23 g/g) compared with the bare EG (110 g/g). Besides, Wang et al. reported the preparation of expanded graphite modified with CoFe_2_O_4_ via sol-gel followed by the EG/CoFe_2_O_4_ weight ratio of 2:1 [11]. It was found that the specific surface area and saturation magnetization of EG-CoFe_2_O_4_ were 216.28 m^2^/g and 17.94 emu/g, respectively. The adsorption capacity of EG-CoFe_2_O_4_ was found to be 48.93 g/g, 42.75 g/g, and 33.18 g/g, respectively, for engine oil, crude oil, and diesel oil. The study by Vinh et al. (2018) on the composites of EG and NiFe_2_O_4_ showed the magnetism value and surface area of 14.2 emu/g and 32.9 m^2^/g, respectively, with high sorption capacity for diesel oil at 32.56 g/g [15]. The latter investigations improved the saturation magnetization of EG by introducing the CoFe_2_O_4_ and NiFe_2_O_4_ up to 42 emu/g and 19 emu/g, respectively [13]. Furthermore, owing to a large number of macropores in the structure, the prepared EG-CoFe_2_O_4_ and EG-NiFe_2_O_4_ proved to have high sorption capacity toward oil of 45–52 g/g. Recently, Ivanov et al. reported the modification of expanded graphite with MgFe_2_O_4_ via co-precipitation method [16]. It was found that the low magnetism value of as-prepared EG-MgFe_2_O_4_ was documented at 16.1 emu/g, whereas the high uptake for crude oil was 54 g/g. Based on documented findings, it should be noticed that the spinel ferrites with formula MFe_2_O_4_ (M = Ni, Co, Mg) have drawn considerable attention on the introduction on EG due to their magnetic property, chemical stability, and biocompatibility.

The present work reports the synthesis and characterization of EG modified with MnFe_2_O_4_ via sol-gel technique applying to eliminate the oil-based pollutants in aqueous solution. Based on the available literature, the addition of MnFe_2_O_4_ particles into EG has not been studied before. Herein, the EG-MnFe_2_O_4_ was prepared via facile two-stage process, including the exfoliation of graphite sheets, using environmentally friendly oxidant agent, namely, hydrogen peroxide, and the incorporation of MnFe_2_O_4_ on graphitic layers using the sol-gel technique. Subsequently, the morphology, structure, and the magnetic properties of the as-prepared EG-MnFe_2_O_4_ were studied by using FTIR, SEM, XRD, Energy-Dispersive X-ray (EDS), Vibrating Sample Magnetometer (VSM), and nitrogen adsorption/desorption analysis. The as-prepared composites were then applied to remove diesel oil and crude oil with varying influential factors, namely contact time, oil dosage, and salt concentration.

## 2. Experimental

### 2.1. Materials and Methods 

Sulfuric acid (H_2_SO_4_, 98%), hydrogen peroxide (H_2_O_2_, 30%), ammonia solution (NH_3_.H_2_O, 25%), citric acid (C_6_H_8_O_7_.H_2_O), Iron (III) nitrate (Fe(NO_3_)_3_.9H_2_O), manganese chloride (MnCl_2_.4H_2_O), sodium hydroxide (NaOH) were provided by Xilong Chemical Co., Ltd., Shantou, China. Diesel oil and crude oil, purchased from PV Oil Co., Ltd., (Ho Chi Minh, Vietnam), were used as received. Natural graphite flakes with carbon content 85–93% and average particle size of about 0.125 mm were collected from Mau A graphite mine located in Yen Bai province of Vietnam. Expanded graphite (EG) was prepared by using H_2_O_2_ and H_2_SO_4_ as oxidizing and intercalating agents under microwave assisted heating at 750 W for 10 s [17].

### 2.2. Preparation of EG-MnFe_2_O_4_

The MnFe_2_O_4_–decorated EG was prepared via citric acid-based sol-gel process. The aqueous solution of MnCl_2_ 4H_2_O and (Fe(NO_3_)_3_.9H_2_O) with a molar ratio of Mn^2+^:Fe^3+^ as 1:2 was vigorously stirred until complete dissolution of the metal salt mixture was obtained. The citric acid (molar ratio of C_6_H_8_O_7_:Fe^3+^ as 4:1) was then added in the mixture under magnetic stirring at the temperature of 90 °C. Subsequently, the addition of EG into solution was performed following the weight ratio between EG and MnFe_2_O_4_ of 85%:15%. The solution pH was adjusted to 9 by gradually adding an ammonia solution to the mixture. After that, the solution was continuously stirred under the temperature of 80 °C until it was concentrated and subsequently dried at 80 °C in the oven for 24 h. Finally, the dried solids were calcined at the temperature of 700 °C for 2 h to obtain EG-MnFe_2_O_4._

### 2.3. Characterization

In order to determine chemical interaction between EG and MnFe_2_O_4_, the Fourier transform infrared (FTIR) spectra were recorded in the range of 4000−400 cm^−1^ on a Bruker Alpha spectrophotometer (Bruker Japan K.K., Tokyo, Japan) using KBr powder. The morphology of EG and EG-MnFe_2_O_4_ was examined using a Scanning Electron Microscope (SEM) on the S4800 equipment (Hitachi High-Tech Solutions Corp., Fukuoka, Japan). The phase composition of EG with and without MnFe_2_O_4_ was analyzed by X-ray diffraction (XRD) patterns on a Bruker D8 Advance powder diffractometer (Bruker Japan K.K., Tokyo, Japan), using Cu-K as radiation source with a scan rate of 0.2/s in the 2-theta range of 2–60°. The elemental components of material were determined by using energy dispersive X-ray spectrometry (EDS) on Oxford instrument. The saturation magnetization of EG-MnFe_2_O_4_ was measured on a GMW 3474-140 magnetometer (GMW associates, San Carlos, CA, USA). The textural properties of the samples were determined using the N_2_ adsorption/desorption measurement on a Tristar II Plus equipment (Micromeritics Instrument Corp., Norcross, GA, USA).

### 2.4. Adsorption Evaluation of EG-MnFe_2_O_4_

The adsorption experiment of EG-MnFe_2_O_4_ for diesel oil and crude oil was performed by adding 0.2 g of EG-MnFe_2_O_4_ to a certain mixture of heavy oil and water. After a target exposure time, the EG-MnFe_2_O_4_ containing oil floating on the surface was collected using a magnetic bar and weighted on the analytical balance. The effect of uptake conditions, namely, expose time, mixing oil dosage, and salt concentration, on the adsorption capacity of EG-MnFe_2_O_4_ was investigated. Each experiment was repeated three times to get the average data. The adsorption capacity (g/g) was calculated using the following equation:

$$\mathrm{Adsorption}\;\mathrm{capacity}=\frac{m_{i}-m_{e}}{m_{i}}$$

where m_i_ (g) and m_e_ (g) are the weight of EG-MnFe_2_O_4_ before and after oil adsorption respectively.

## 3. Results and Discussion

### 3.1. SEM and EDS analysis

**Figure 1:**
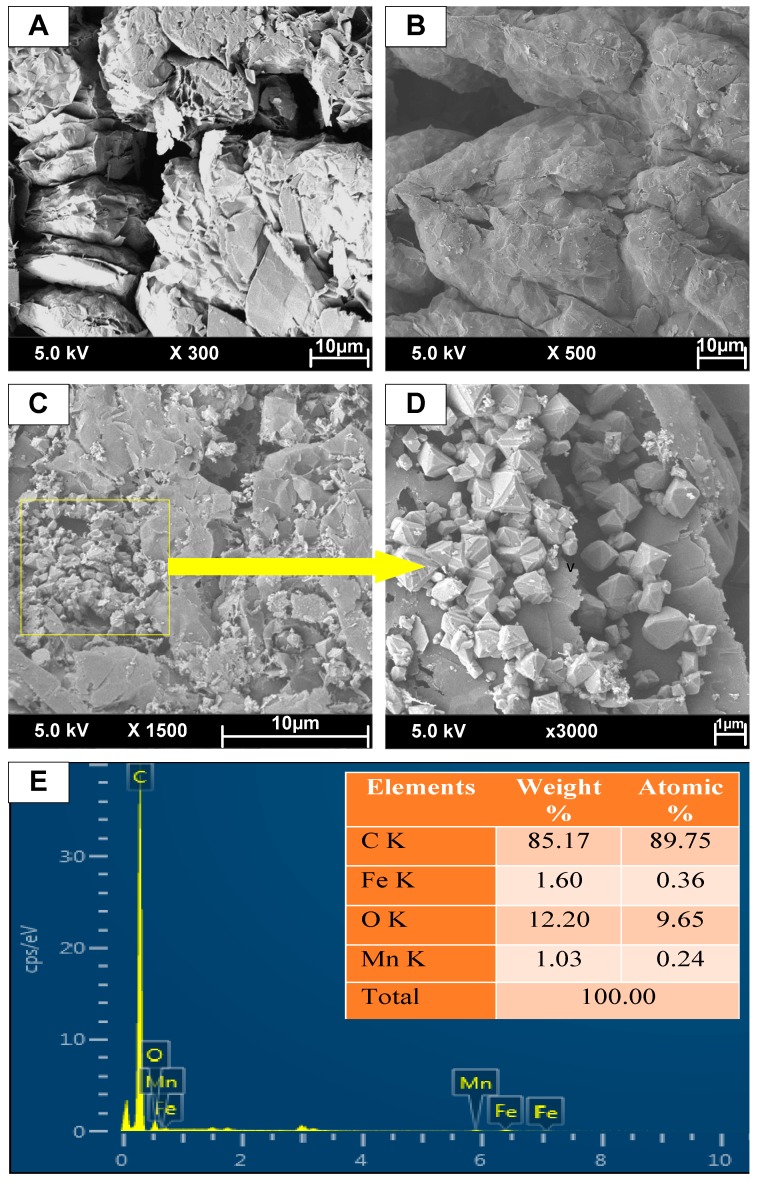
SEM images of expanded graphite (EG) (**A**), EG-MnFe_2_O_4_ (**B**), details of EG-MnFe_2_O_4_ (**C**,**D**), and Energy-Dispersive X-ray (EDS) of EG-MnFe_2_O_4_ (**E**).

The worm-like structure and the plenty of the capillary interstices were clearly observed for expanded graphite (EG) as seen in Figure 1A. The SEM image of EG-MnFe_2_O_4_ is presented in Figure 1B. After the modification with MnFe_2_O_4_, the worm-like structure of EG was not remarkably different compared to EG-MnFe_2_O_4_; however, the length of worm-like particles in the as-prepared EG-MnFe_2_O_4_ composites was expanded and developed in comparison with those in EG. It can be seen from Figure 1C that MnFe_2_O_4_ particles were distributed on the surface unevenly and occupied partly on the external side of pores and nanocytes. For more details, the MnFe_2_O_4_ particles were observed in the octahedral structure (Figure 1D). The SEM results elucidated the successful decoration of MnFe_2_O_4_ on the graphite sheets. The presence of Mn, Fe, and O elements beside the primary ingredient of C in the as-fabricated material implied the successful incorporation of MnFe_2_O_4_ into graphite layers.

### 3.2. FTIR Analysis

**Figure 2:**
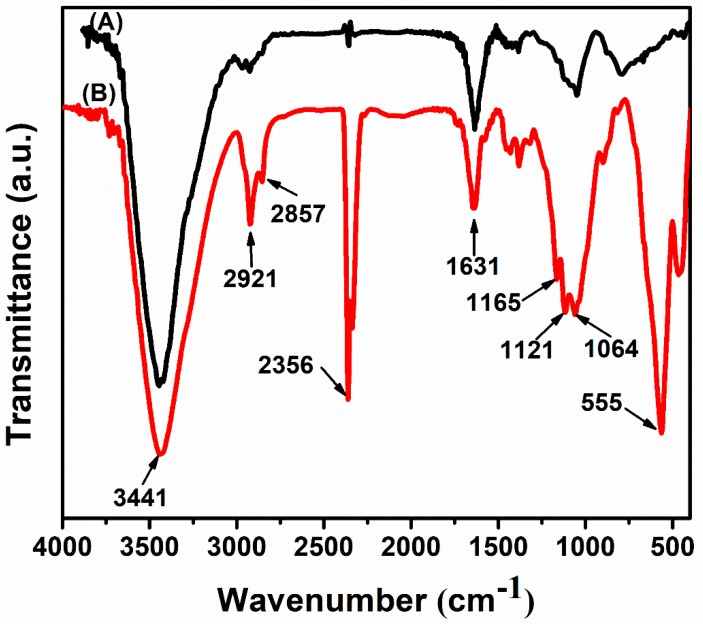
FTIR analyses of expanded graphite (EG) (**A**) and EG-MnFe_2_O_4_ (**B**).

FTIR spectroscopy was used to determine the structural interaction between expanded graphite (EG) and MnFe_2_O_4_ as observed in Figure 2. For EG spectrum, the characteristic peaks located at 3441 cm^−1^ and 1631 cm^−1^ were assigned to the stretching and bending vibration of O-H groups; at 2921 and 2857 cm^−1^, showed the stretching vibration of C-H in CH, CH_2_, and CH_3_; at 2356 cm^−1^, were related to CO_2_ stretching vibration; at 1050 cm^−1^, could be attributed to C-O-C symmetric vibration [18,19]. After decoration with MnFe_2_O_4_, some new peaks appeared at 1165 and 1121 cm^−1^, which were assigned to bonded hydroxyl groups on the metal, and at 555 cm^−1^, which showed intrinsic stretching vibration of Mn-O bonds characterized for octahedral manganese ferrite [20,21,22]. These results demonstrate the existence of MnFe_2_O_4_ on EG.

### 3.3. XRD and VSM Analysis

**Figure 3:**
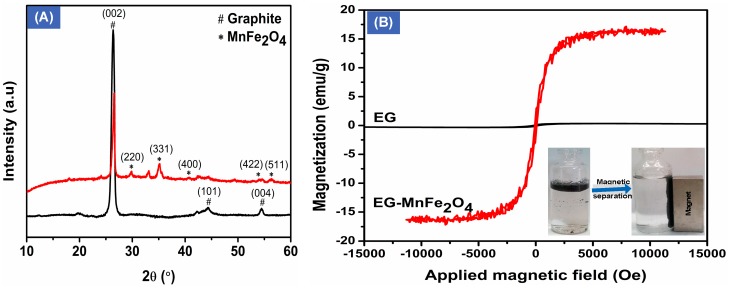
XRD spectra (**A**) and Vibrating Sample Magnetometer (VSM) analyses (**B**) of expanded graphite (EG) and EG-MnFe_2_O_4_.

Figure 3A shows the XRD spectra of EG and EG-MnFe_2_O_4_. As can be observed, the main diffraction peaks were found for EG on the ground of the different phases of the carbon-based material. The peaks at 26.43°, 44.32°, and 54.54°, respectively, are corresponding to hexagonal lattice of (002), (101), and (004) planes of graphite, respectively [12,14]. The emergence of new peaks with the addition of MnFe_2_O_4_ into EG was also observed in the XRD spectrum. For instance, the appearance of peaks at 2θ = 30.10°, 35.50°, 43.10°, and 57.00°, respectively, stands for the (220), (311), (400), and (511) reflections of octahedral MnFe_2_O_4_ [23,24], indicating the successful incorporation of magnetic particles to graphite layers. Additionally, the disappearance of peak at 44.32° and 54.54° in the XRD spectrum of modified EG indicated that graphite sheets were further expanded during the synthesis process with heating at 700 °C.

The magnetic properties of EG and EG-MnFe_2_O_4_ were studied using VSM analyses. It can be seen from Figure 3B that the saturation magnetization of EG is close to zero. With additional magnetic octahedralMnFe_2_O_4_ particles into EG, the value of saturation magnetization was found to be 16 emu/g, which is much lower than the theoretical magnetism value of bare MnFe_2_O_4_ reported in the literature [23,24]. This is given that the MnFe_2_O_4_ wrapped by non-magnetic graphite sheets presenting as antiferromagnetic layers on the surface may result in a decrease in magnetism of EG-MnFe_2_O_4_. Some similar findings showed weak magnetism of expanded graphite containing MgFe_2_O_4_ (16.1 emu/g), reported by Ivanov et al. [16]. However, the incorporation of NiFe_2_O_4_ and CoFe_2_O_4_ exhibited higher value at 19 and 40 emu/g, respectively [13]. This might be attributed to the size, sharpness, and quantity of decorated ferrite particles on the EG. Noticeably, albeit weak, the addition of MnFe_2_O_4_ into graphite layers significantly improved the magnetism of EG, allowing the easy and rapid collection of absorbent after use under the magnetic field. The obtained results well-agreed with the SEM, EDS, and XRD analyses that octahedral MnFe_2_O_4_ was successfully added into EG.

### 3.4. N_2_ Adsorption/Desorption Analyses

**Figure 4:**
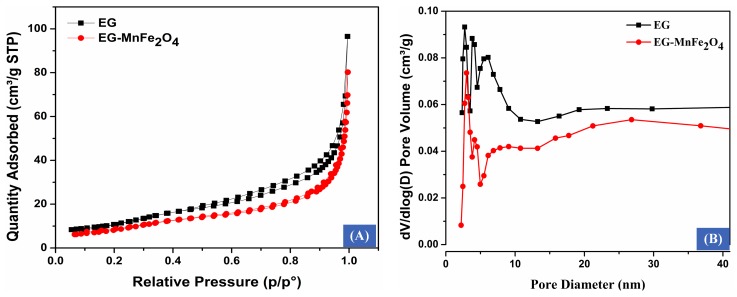
The N_2_ adsorption/desorption isothermal of MnFe_2_O_4_-decorated expanded graphite (**A**) and the corresponding distribution of the pore diameter (**B**).

Figure 4A reveals N_2_ adsorption/desorption isotherms of EG and EG-MnFe_2_O_4_. It can be seen that the feature hysteresis between desorption and adsorption curve of EG is characterized by the type IV, which demonstrates the presence of a moderate number of mesopores and very little micropores in vermiculate EG [18]. The decoration of MnFe_2_O_4_ on graphite layers induced a little decline in the number of mesopores and micropores when the narrower isotherm features hysteresis between desorption and adsorption curve on EG-MnFe_2_O_4_. This was also exhibited in the pore diameter distribution curves of bare EG and EG-MnFe_2_O_4_ in the range of approximately 2−10 nm (Figure 4B). The Brunauer-Emmett-Teller (BET) surface area, pore diameter, and cumulative pore volume of graphite-based adsorbents are presented in Table 1. The results exhibited specific surface area and pore volume of EG at 41 m^2^/g and 156.10^−3^ cm^3^/g, respectively. With the addition of MnFe_2_O_4,_ these parameters of EG-MnFe_2_O_4_ were documented at 32 m^2^/g and 156.10^−3^ cm^3^/g, respectively, slightly decreased values compared with EG. This might be attributed to the distribution of MnFe_2_O_4_ particles on the surface of EG and, as a result, there is a decrease in the total surface area and cumulative pore volume of EG-MnFe_2_O_4_ in comparison with EG [11]. The obtained results indicated that the added manganese ferrite had a minor effect on the texture of EG-MnFe_2_O_4_.

### 3.5. The Adsorption of EG-MnFe_2_O_4_ for Heavy Oil

**Figure 5:**
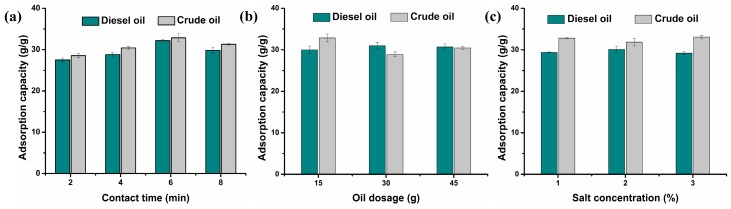
The effect of contact time (**a**), oil dosage (**b**), and salt concentration (**c**) on the adsorption capacity of EG-MnFe_2_O_4_ for crude and diesel oil.

The adsorption capacity of EG-MnFe_2_O_4_ was investigated for crude oil (CO) and diesel oil (DO). Figure 5a illustrates the time exposed between adsorbents and adsorbates to determine the sorption rate. It was found that the maximum adsorption capacity of EG-MnFe_2_O_4_ was reached within 6 minutes for CO and DO. Furthermore, the sorption amount of EG-MnFe_2_O_4_ for crude oil (33.07 g/g) is higher than for diesel oil (32.20 g/g). The reason may be due to the larger glutinosity of crude oil, resulting in the higher density of oil molecules occupied inside pores [15]. Comparably, the adsorption amount of bare EG for CO and DO was found to be 47.7 g/g and 39.8 g/g within 4 minutes, respectively. These results showed that the MnFe_2_O_4_ insertion decreased the adsorption capacity of EG but still possessed a significant adsorption ability for practical application. Regarding the practical utilization of magnetic graphite-based materials for treatment on the large scale environment, namely, ocean, where oil spill accidents happen, both the salinity and oil dosage may considerably affect the adsorption efficacy. Figure 5b presents the possible impact of water toward the oil amount loading on the water surface. It can be seen that adsorbed DO capacity did not change considerably while CO uptake caused small fluctuation in oil dosage ranging from 15 gg to 45 g. The effect of salinity was studied in the marine environment on the adsorption performance of EG-MnFe_2_O_4_ for DO and CO with a fixed oil content of 15 g per 30 g H_2_O and NaCl concentration of 1–3% (Figure 5c). The obtained results showed that the variation in the NaCl concentration just exerted slight fluctuation in the adsorption capacity of EG-MnFe_2_O_4_ for CO and DO, which indicated the efficient removal of heavy oil in the saline water environment. In brief, it can be concluded that the EG-MnFe_2_O_4_ hybrid materials can be considered as a promising constituent for the treatment of heavy oil in the real marine environment.

## 4. Conclusions

Based on obtained results, one can conclude that the magnetic MnFe_2_O_4_-decorated EG material was successfully fabricated from Vietnamese graphite flakes via facile two-step route. The SEM images and FTIR, XRD, EDS, and VSM analyses indicated the presence of MnFe_2_O_4_ octahedral on graphite layers. The magnetism value and specific surface area of EG-MnFe_2_O_4_ were found to be 16 emu/g and 32 m^2^/g, respectively. The adsorption results revealed the excellent capture of as-prepared EG-MnFe_2_O_4_ toward heavy oils within a short time. The adsorption amount of DO and CO on EG-MnFe_2_O_4_ was found to be 32.20 g/g and 33.07 g/g, respectively. The experimental conditions, namely, oil dosage and salt concentration, did not significantly affect the adsorption capacity of EG-MnFe_2_O_4_ for diesel and crude oil. Briefly, the successful synthesis of MnFe_2_O_4_-decorated EG is a potential and promising technology for the application in water purification.

## Figures and Tables

**Table 1:** The textural properties of expanded graphite (EG) and EG-MnFe_2_O_4_.

Adsorbent	BET Surface Area (m^2^/g)	Brunauer-Joyner-Halenda (BJH) Desorption Average Pore Diameter (4V/A) (nm)	BJH Desorption Cumulative Pore Volume (cm^3^/g)(17–3000 A^0^)
**Exfoliated graphite (EG)**	41.0	12.0	156.10^−3^
**EG-MnFe_2_O_4_**	32.0	14.0	130.10^−3^
